# Context-Dependent cGAS-STING Activation Shapes Metastatic Progression and Dormancy

**DOI:** 10.3390/cells15171619

**Published:** 2026-09-05

**Authors:** Eilma Akter-Yasuoka, Tak Lee, Takahiko Murayama, Jun Nakayama

**Affiliations:** 1 Division of Cell Signaling and Informatics, Cancer Research Institute, Kanazawa University, Kanazawa 920-8640, Japan; eilma@oici.jp; 2 Bioinformatics and Omics Unit, Collaborative Research Promotion Domain, Next-Generation Precision Cancer Research Center, Osaka International Cancer Institute, Osaka 541-8567, Japan; 3 Department of Oncogenesis and Growth Regulation, Frontier Exploratory Research Domain, Next-Generation Precision Cancer Research Center, Osaka International Cancer Institute, Osaka 541-8567, Japan; 4 Department of Cell Biology, SUNY Downstate Health Sciences University, Brooklyn, NY 11203, USA; tak.lee@downstate.edu

**Keywords:** cGAS-STING, metastasis, dormancy, tumor microenvironment

## Abstract

**Highlights:**

**What are the main findings?**

- cGAS-STING signaling has context-dependent roles in metastasis.
- Dormant disseminated tumor cells repress STING signaling and related immune ligands in perivascular niches, enabling immune evasion and long-term persistence.

**What are the implications of the main findings?**

- Temporal, cell-type, and spatial control of STING activation are critical for STING-targeted therapies and biomarker development.
- Rational combination strategies are needed to harness acute STING-mediated immunity while avoiding chronic, tumor-promoting inflammation.

**Abstract:**

Cancer cells survive, proliferate, and metastasize in part because the immune system fails to detect and eliminate them. Moreover, the tumor microenvironment (TME) that surrounds the tumor supports cancer cell survival and resistance to chemo- and immunotherapies by inhibiting antitumor immune responses and thereby reducing the efficacy of immunotherapeutic interventions. cGAS-STING signaling senses cytoplasmic DNA and coordinates innate immune responses that shape tumor-intrinsic outcomes and the TME. Emerging evidence reveals a context-dependent, dualistic role for cGAS-STING in metastatic progression and cancer dormancy. Acute, robust activation in antigen-presenting cells promotes type I interferon responses, leading to suppression of tumor growth. By contrast, chronic, low-level cancer-intrinsic STING signaling can engage inflammatory programs that foster immune suppression and therapy resistance. Dormant disseminated tumor cells exploit niche cues to downregulate STING signaling and evade immune detection, whereas reactivation of dormant cells often involves restoration of STING activity that can promote immune elimination. In this article, we review mechanisms linking genome instability and cytoplasmic DNA to STING activation, summarize evidence for tumor-suppressive versus tumor-promoting functions across metastatic niches, and discuss how STING agonists and combination strategies may be optimized to maximize antitumor immunity while avoiding protumorigenic effects.

## 1. Introduction

Cancer cells acquire uncontrolled proliferative capacity, invade surrounding tissues, and eventually disseminate to distant organs to form metastases [1,2]. Since metastasis involves multiple cellular and environmental stresses, successful metastatic colonization depends on interactions between disseminated tumor cells (DTCs) and the microenvironment of distant organs [3,4]. During metastasis, epithelial–mesenchymal transition (EMT) promotes cancer cell dissemination by enhancing migratory and invasive properties [5,6]. Following intravasation, circulating tumor cells (CTCs) travel through the bloodstream and seed distant metastatic sites [7]. Extravasation of CTCs requires transendothelial migration (TEM) across the endothelial barrier to enter distant tissues [8]. CTCs are exposed to various physical and immune stresses during circulation, including shear stress in the bloodstream and NK cell-mediated attack [9,10]. Following extravasation, DTCs encounter unfamiliar microenvironments in distant organs, and many fail to survive or establish metastatic lesions [11]. To survive in distant organs, some DTCs enter a dormant state to evade immune surveillance and adapt to the metastatic microenvironment. Cancer cell dissemination has been shown to occur not only in advanced tumors but also during the early stages of tumor development [12]. Notably, early disseminated cancer cells are prone to enter a dormant state [13,14]. Cancer dormancy is characterized by a prolonged state of growth arrest in which disseminated tumor cells persist in distant organs without forming clinically detectable metastases [15]. Dormant cancer cells can survive in a reversible G0-like state with reduced metabolic activity for years or even decades before re-entering the cell cycle and causing metastatic recurrence [16]. Dormant DTCs frequently localize to perivascular niches in distant organs, where the microenvironment supports their survival and maintains growth arrest [17]. Consistently, dormant DTCs have been observed in perivascular niches in multiple organs, including the brain, suggesting a critical role for the perivascular microenvironment in sustaining metastatic dormancy [18].

During metastatic colonization, cancer cells are exposed to diverse intrinsic and extrinsic stresses, including oxidative stress, replication stress, and immune surveillance [19]. These cellular stresses can induce DNA damage and chromosomal instability (CIN). Genome instability is one of the hallmarks of cancer, and elevated mutation burdens associated with defective DNA repair contribute to various hereditary and sporadic cancers [20]. During cancer progression, chronic hypoxic stress in the process of cancer metastasis might also contribute to genomic instability and deregulate DNA-damage response pathways [21]. In addition, oncogene activation and subsequent replication stress promote double-strand break formation, thereby fueling CIN [22]. CIN has been recognized as a feature of many tumors and it is associated with poor prognosis, metastasis, and therapeutic resistance [23,24]. Furthermore, genome instability and CIN lead to the generation of cytosolic DNA that activates the cGAS-STING pathway.

The cGAS-STING pathway plays a central role in host defense against microbial infections as well as in antitumor immunity, cellular senescence, autophagy, and autoimmune and inflammatory diseases, owing to its ability to recognize double-stranded DNA in a sequence-independent manner [25,26]. When DNA is present in the cytoplasm, cGAS binds to it, inducing a conformational change in the active site. This conformational change catalyzes the synthesis of cyclic GMP-AMP (cGAMP) from ATP and GTP. cGAMP functions as a second messenger that binds to the endoplasmic-reticulum (ER)-membrane adaptor STING and induces a conformational change that results in STING activation [27]. STING is a ∼42 kDa protein composed of an N-terminal four-transmembrane domain associated with the ER membrane and a C-terminal ligand-binding domain (LBD) followed by a C-terminal tail facing the cytosol. Upon cGAMP binding to STING, the LBD wings rotate, resulting in closure of the central pocket via a four-stranded β-sheet lid. This conformational change leads to an expanded conformation of the transmembrane domain and the side-by-side assembly of STING dimers into oligomers [28]. Activated STING dimers translocate to the trans-Golgi network through COP-coated vesicle, where Cys88 and Cys91 of STING are palmitoylated [29,30,31]. Palmitoylated STINGs form clusters as a scaffold of its downstream effector TANK-binding kinase 1 (TBK1), facilitating TBK1 trans-autophosphorylation [32,33,34]. Phosphorylated TBK1, in turn, phosphorylates adjacent STING molecules at multiple sites, which serve as a hallmark of STING activation. Phosphorylated STING oligomers act as a platform that recruits the transcription factor interferon regulatory factor 3 (IRF3), leading to IRF3 phosphorylation, dimerization, and nuclear translocation [35]. Additionally, activation of STING leads to NF-κB nuclear translocation via recruitment of IKK kinases, which induce the expression of interferons (IFNs) and proinflammatory cytokines, including TNF, IL-1, and IL-6 [36]. To prevent excessive inflammatory responses, STING further moves to recycling endosomes. Activated STINGs at recycling endosomes are polyubiquitinated and terminated through transport-dependent lysosomal microautophagy, which mediates its degradation at the lysosomal membrane [37,38].

Nucleic acid sensing is a fundamental mechanism for detecting microbial pathogens and maintaining innate immune homeostasis [39]. Among these pathways, the cGAS–STING axis functions as a central cytosolic DNA-sensing system and has recently been implicated in a variety of autoinflammatory diseases as well as cancer [40]. Given that metastatic dissemination and cancer dormancy are accompanied by persistent cellular stress, genomic instability, and cytosolic DNA accumulation, the cGAS-STING pathway has emerged as a critical regulator of metastatic progression, dormancy, and the interactions between disseminated tumor cells and the metastatic microenvironment. In this review, we summarize the current understanding of the multifaceted roles of cGAS-STING signaling in metastasis and cancer dormancy, with a particular focus on its context-dependent functions in tumor progression and immune regulation.

## 2. Cancer Metastasis and STING

### 2.1. Tumor-Suppressive Functions of cGAS-STING Signaling

Activation of the cGAS-STING pathway in tumor cells may suppress early tumor progression through the induction of type I IFNs and other inflammatory genes [41]. Loss of STING signaling has been associated with increased tumor incidence. In agreement with this, reduced STING expression has been observed in several melanoma and colorectal adenocarcinoma cell lines [42,43]. Importantly, the cGAS-STING pathway has been strongly implicated in the induction of cancer cell senescence, thereby exerting tumor-suppressive effects [44]. cGAS-STING-mediated senescence is associated with the production of senescence-associated secretory phenotype (SASP) factors, including chemokines, proinflammatory cytokines, growth factors, and proteases [45]. These immune-stimulatory factors can promote tumor suppression either through tumor cell-intrinsic mechanisms or by activating antitumor immune responses [46]. In addition, activation of the STING pathway promotes T cell infiltration into tumors by inducing chemokine expression and normalizing the tumor vasculature [36]. The cGAS-STING pathway enhances antitumor immunity by promoting both T cell- and NK cell-mediated tumor eradication. In several cancers, including melanoma, suppression of cGAS-STING signaling impairs MHC class I expression and facilitates immune evasion, whereas activation of STING restores antigen presentation and enhances cytotoxic T lymphocyte (CTL)-mediated tumor killing [47]. In addition, STING activation promotes the recruitment and activation of cytotoxic T cells and NK cells through the induction of chemokines such as CXCL9, CXCL10, and CCL5, thereby facilitating antitumor immune responses [48,49,50]. Genome instability frequently induces micronuclei formation in cancer cells, leading to cGAS activation and STING-dependent tumor-suppressive immune responses. These findings suggest that cGAS-mediated sensing of micronuclei functions as a cell-intrinsic surveillance mechanism linking genome instability to innate immunity [51].

### 2.2. Protumorigenic Roles of cGAS-STING Signaling

Although the cGAS-STING pathway is widely recognized as a mediator of antitumor immunity, accumulating evidence suggests that STING signaling can paradoxically promote tumor progression [52]. For example, reduced or absent STING expression in small-cell lung cancer (SCLC) compromises type I IFN signaling and adaptive immunity [53]. In contrast, persistent activation of the cGAS-STING pathway can be exploited by tumors to promote tumor growth, metastasis, drug resistance, and immune suppression [54]. DNA damage induced by carcinogens can lead to cytosolic DNA accumulation and activation of STING-dependent innate immune signaling, thereby contributing to inflammation-associated tumorigenesis [55]. Chronic activation of the cGAS-STING pathway promotes immune suppression by inducing T cell exhaustion and recruitment of immunosuppressive cells, including Tregs, MDSCs, and Bregs. In addition, persistent STING signaling upregulates immunosuppressive molecules and preferentially activates noncanonical NF-κB signaling, thereby facilitating tumor progression [56,57,58]. Activation of noncanonical NF-κB signaling promotes tumor cell survival, proliferation, and inflammatory responses. In highly metastatic tumors, cancer cells can transfer cGAMP to stromal cells, activating STING signaling and inducing inflammatory cytokine production, which in turn enhances tumor survival, proliferation, and chemoresistance through STAT1 and NF-κB signaling [59]. In addition, oncogenic viruses such as HPV suppress cGAS-STING signaling to evade innate immunity [60]. Collectively, these findings suggest that chronic or dysregulated cGAS-STING signaling can remodel the tumor microenvironment and promote malignant phenotypes that support tumor progression and metastasis.

## 3. Cancer Cell Dormancy and STING

### 3.1. STING Signaling and Metastatic Dissemination

The cGAS-STING pathway is a critical innate immune pathway that influences multiple stages of tumor progression, including metastasis [61]. CIN-induced micronuclei activate chronic cGAS-STING signaling in cancer cells, preferentially inducing noncanonical NF-κB signaling rather than type I IFN responses. This pathway promotes EMT, invasion, and metastatic dissemination, highlighting a protumorigenic role of chronic STING activation [62]. Esophageal adenocarcinoma is also characterized by CIN, which drives chronic cGAS-STING activation and chemokine-mediated recruitment of protumor inflammatory infiltrates, thereby fostering tumor survival and metastasis [63]. In the case of the brain, metastatic cells selectively establish Cx43-mediated gap junctions with astrocytes through protocadherin 7 (PCDH7), enabling the transfer of cGAMP from cancer cells to astrocytes and subsequent activation of the STING pathway. STING activation in astrocytes induces the production of IFN-α and TNF-α, which promote the growth and chemoresistance of brain metastatic cells [64]. Given the prometastatic functions of chronic STING activation, tight regulation of STING signaling appears to be critical during tumor progression. In colorectal cancer, SEL1L3 inhibition stabilizes STING signaling by endoplasmic reticulum-associated degradation, thereby suppressing growth and migration [65]. Collectively, these findings indicate that chronic cGAS-STING signaling promotes metastatic progression through both tumor cell-intrinsic mechanisms and remodeling of the metastatic microenvironment.

### 3.2. Cellular Stress in Metastatic Dormancy

Metastatic cancer cells are exposed to multiple stresses during dissemination, dormancy, outgrowth, and therapy resistance, and only a small fraction ultimately establish distant metastases [66]. Following extravasation, disseminated cancer cells encounter oxidative stress, nutrient deprivation, and insufficient growth signals, while tissue resident immune cells, including macrophages, NK cells, and T cells, actively suppress metastatic outgrowth. Under these conditions, disseminated cancer cells survive in a quiescent state until they are either eliminated or eventually resume proliferative growth [67]. Therefore, cancer cells enter a dormant state to escape from physical, metabolic, and immune barriers [68]. Recent studies have revealed that dormant metastatic cells undergo a distinct TGFβ-induced epithelial–mesenchymal transition (EMT) program that differs from the classical invasive EMT observed in proliferating metastatic cells [69]. In the perivascular niche, TGFβ induces a reversible quiescent state accompanied by suppression of MHC-I molecules, NK cell-activating ligands, and STING signaling, thereby enabling dormant cancer cells to evade immune surveillance [70]. Mechanistically, dormant metastatic progenitors adopt an atypical EMT state lacking actin stress fibers through TGFβ-dependent expression of the actin-depolymerizing protein gelsolin, resulting in reduced cell stiffness and escape from immune mechanosurveillance. These findings suggest that EMT plasticity contributes not only to metastatic dissemination but also to immune evasion and long-term survival during metastatic dormancy [69].

### 3.3. STING Signaling Suppresses Metastatic Outgrowth

**Figure 1:**
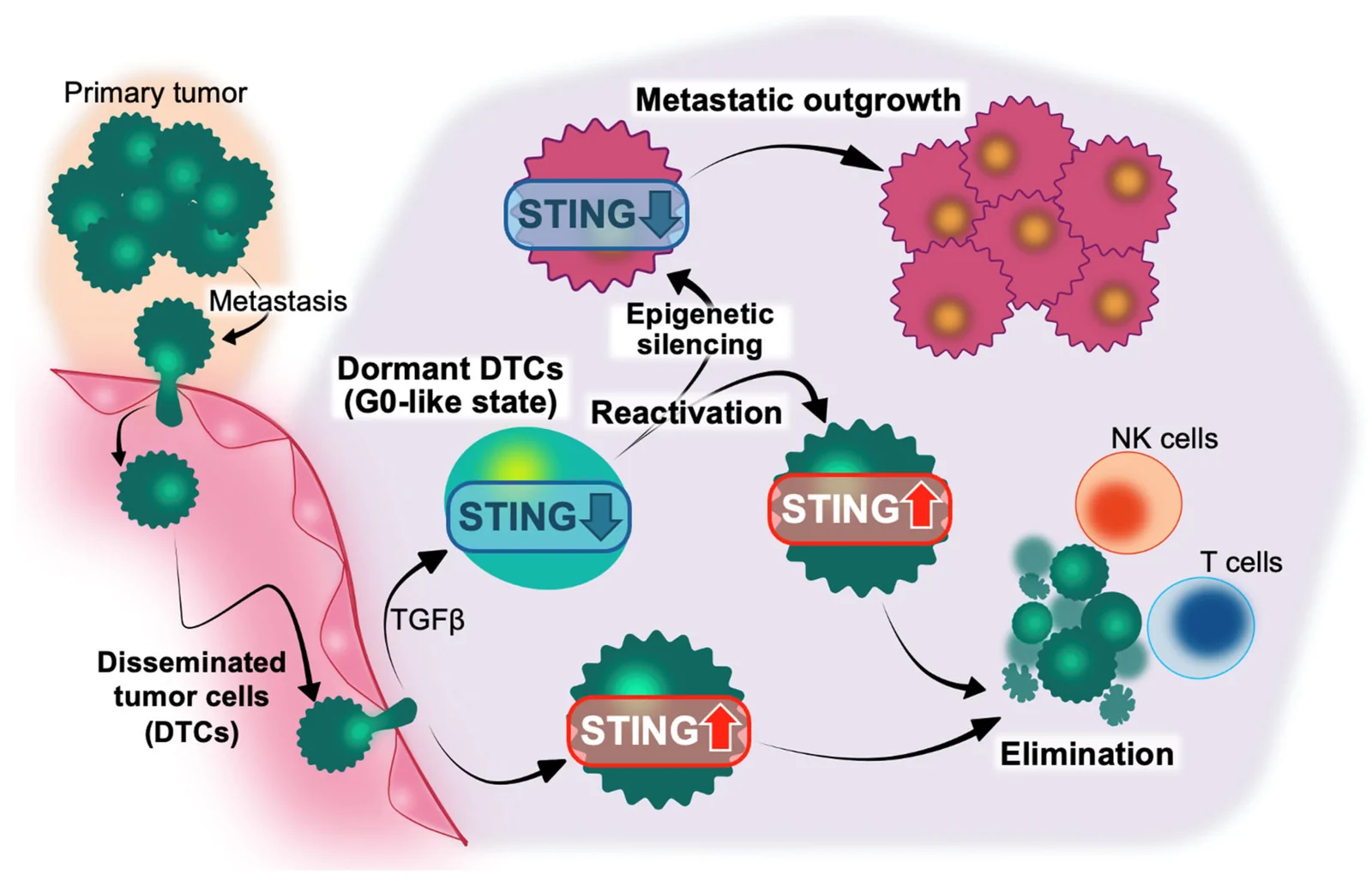
STING activity during metastatic dissemination and cancer dormancy.

Immune surveillance has emerged as a critical barrier that prevents dormant cancer cells from resuming metastatic outgrowth after cell cycle re-entry [71]. During dormancy, quiescent cancer cells evade immune surveillance by downregulating NK cell-activating ligands and MHC-I molecules, thereby reducing recognition by NK cells and T cells [72]. The cGAS-STING pathway functions as a suppressor of metastatic reactivation. STING activity is elevated in metastatic progenitor cells that re-enter the cell cycle and promotes NK cell and T cell infiltration. In contrast, epigenetic silencing of STING through promoter hypermethylation or chromatin repression impairs NK cell migration and facilitates metastatic outgrowth. These findings suggest that STING signaling functions as a checkpoint against dormant metastatic progression (Figure 1) [73]. STING activation enhances antitumor immune surveillance during metastatic dormancy by promoting NK cell and T cell infiltration and activation. Consistently, the STING agonist MSA-2 augments NK cell-mediated cytotoxicity against dormant cancer cells and suppresses metastatic outgrowth in advanced breast cancer [74]. Reactivation of dormant metastatic cells induces STING-dependent type I INF signaling, leading to enhanced immune recognition and elimination by cytotoxic lymphocytes. In contrast, epigenetic silencing of STING enables dormant cancer cells to evade immune surveillance and progress toward overt metastatic colonization, while epigenetic remodeling during the EMT–MET transition may further influence STING activity during dormancy reactivation [73,75]. These findings suggest that metastatic dormancy represents a dynamic equilibrium between immune-mediated elimination and adaptive survival programs in disseminated cancer cells (Figure 1).

## 4. Tumor Microenvironment and STING

### 4.1. Components of the Tumor Microenvironment and Therapy Response

Although cancer research has traditionally focused on genetic and epigenetic alterations of cancer cells themselves which drive malignancy, recent findings have revealed that the tumor microenvironment (TME) greatly influences cancer physiology, particularly response to chemotherapy and immunotherapy [76,77,78]. The TME consists of cancer cells, non-malignant cellular components, and non-cellular elements that surround and interact with cancer cells. Major cellular components include immune cells—such as dendritic cells, macrophages, neutrophils, natural killer (NK) cells, and various T and B lymphocyte subsets—and non-immune stromal cells such as cancer-associated fibroblasts (CAFs), endothelial cells forming the vasculature, and pericytes [76,79]. In addition, non-cellular elements include the extracellular matrix (ECM), soluble mediators (cytokines, chemokines, and growth factors), and metabolites, such as lactate and adenosine [80]. Both cellular and non-cellular components have been reported to contribute to tumor evolution and therapeutic response, underscoring the need to understand the TME as an integrated system [80]. Importantly, the TME is dynamically remodeled by reciprocal signaling between cancer cells and the other components, which could either support immune surveillance or promote immune evasion, angiogenesis, and invasion [81,82]. In terms of response to immune checkpoint blockade (ICB) therapies, in particular, infiltration of immune cells into the TME is a critical factor. In general, “cold” (immune-desert or immune-excluded) tumors are more resistant to ICB therapies than “hot” (immune-inflamed) tumors. Activating innate immune responses in tumors is a promising way to convert cold tumors into hot ones [48]. In this context, activation of the cGAS-STING pathway is one of the potent approaches to induce tumor-intrinsic innate immunity, reshape the TME, and enhance the efficacy of ICB therapies.

### 4.2. Functional Differences of cGAS-STING in Cancer Cells Versus Immune Cells in the TME

The cGAS-STING pathway is an evolutionarily conserved defense mechanism developed against infection of DNA viruses [83]. It senses cytoplasmic double-stranded DNA (dsDNA) and initiates innate immune responses via production of type I IFNs [84]. Recently, it has been revealed that not only dsDNA, but DNA/RNA hybrids derived from unprocessed R-loops in cancer cells can also be ligands for this pathway [85]. Intriguingly, the consequences of cGAS-STING activation may differ between cancer cells and stromal immune cells. In major professional antigen-presenting cells (APCs) such as dendritic cells and macrophages, which strongly express both cGAS and STING proteins, activation of the pathway triggers robust type I IFN production [86]. In these normal cells, genomic DNA is generally stable, unlike cancer cells, so cytoplasmic DNA sensed by cGAS typically originates from exogenous sources. Cancer cells often undergo cell death in the TME because of chromatin instability or hypoxia, releasing ample DNA that can be taken up by APCs [86]. Effective antitumor immunity depends on cross-presentation of tumor antigens by activated APCs to T lymphocytes (CD8+ T cells and CD4+ T cells) [87,88]. In addition to the antigen presentation, the cytokines secreted from APCs also promote the recruitment, differentiation and maturation of T lymphocytes, which leads to the subsequent adaptive immune responses [87].

By contrast, cancer cells show heterogenous cGAS-STING status and responses. A portion of tumors retain functional cGAS and STING molecules; accumulation of cytoplasmic dsDNA or DNA/RNA hybrids, which are derived from damaged nuclear or mitochondrial DNA, activates the cGAS–STING pathway, leading to type I IFN production and cell-autonomous outcomes such as senescence or growth arrest, which could suppress tumor growth and survival [89]. Type I IFNs, as well as a series of cytokines downstream of STING, also recruit and activate NK cells and T lymphocytes in the TME to eliminate cancer cells. Cancer cells with unstable chromatin, face a challenge in which chronic accumulation of cytoplasmic DNA poses a risk to activate the cGAS-STING pathway, even without DNA-damaging agent treatment [51]. Thus, some tumors downregulate or mutate pathway components (commonly cGAS or STING) to suppress robust innate immune responses and evade detection from the immune cells [90]. Notably, chronic low-level cancer-intrinsic cGAS-STING signaling has been linked to persistent inflammation, which can support tumor progression [55].

**Figure 2:**
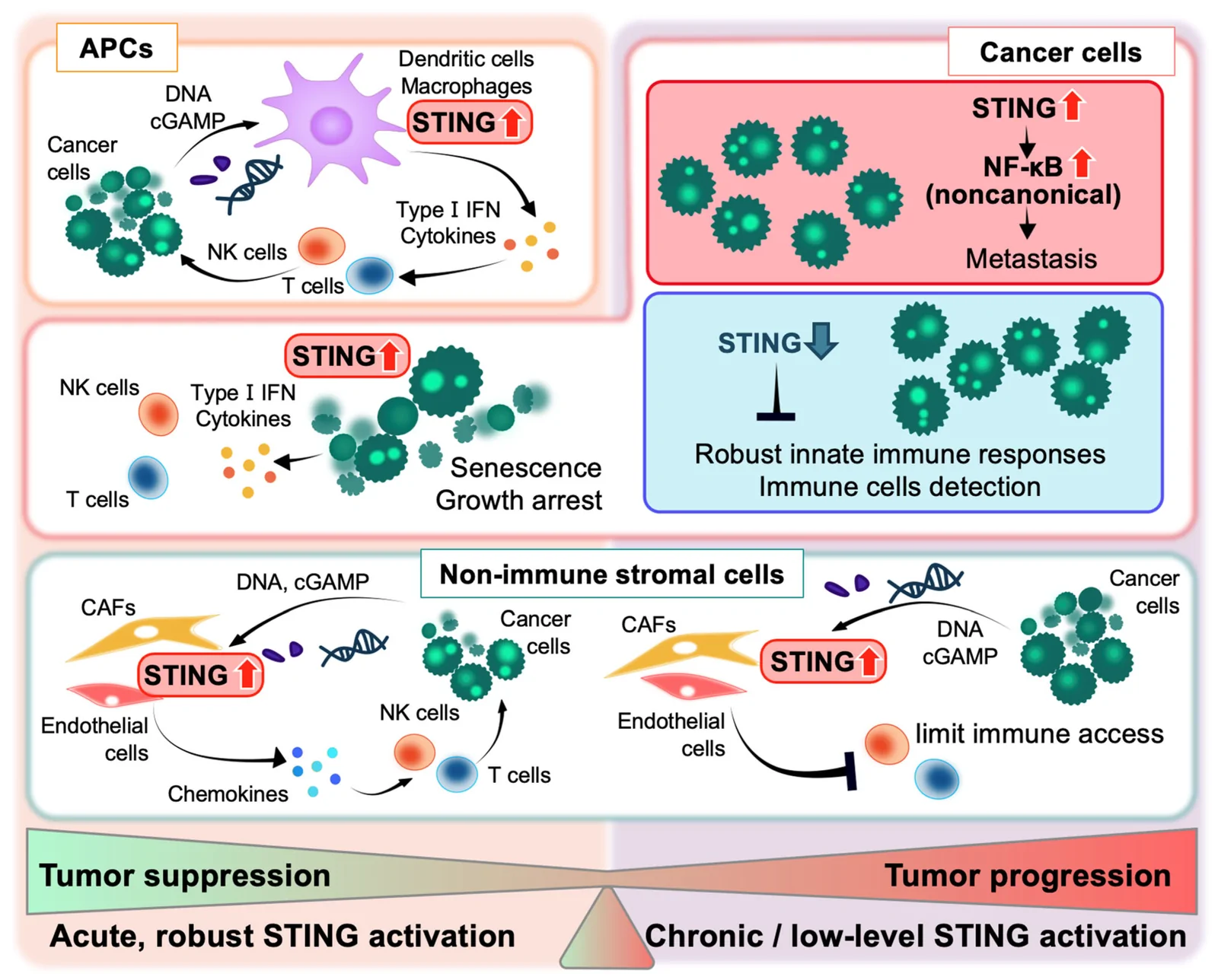
Cell type and activation-dependent functions of STING signaling within the tumor microenvironment.

Moreover, non-immune stromal cells such as cancer-associated fibroblasts (CAFs) and endothelial cells also respond to cGAMP or DNA released from cancer cells. STING activation in these components results in context-dependent outcomes: it can facilitate immune infiltration by inducing type I IFN signaling and T cells attracting chemokines [90,91], or conversely promote protumor functions that limit immune access [89]. These divergent effects may in part reflect the marked heterogeneity and plasticity of stromal cells, particularly CAFS, whose tumor-restraining or tumor-supportive functions vary according to their cellular state, tumor type, and surrounding cytokine and extracellular matrix context [91,92]. Thus, the net effect of cGAS-STING activation in the TME depends on the balance between immune-stimulatory signaling in APCs and potential immunosuppressive or tumor-promoting responses from tumor cells and non-immune stromal compartments (Figure 2).

### 4.3. Tumor-Suppressive Effects and Tumor-Promoting Inflammation Induced by STING Activation

Acute STING activation in the TME is generally tumor-suppressive. When DNA is severely damaged in cancer cells, cancer-intrinsic innate immune responses are induced, or DNA released from dying cancer cells activates APCs, leading to type I IFN secretion that recruits and activates cytotoxic CD8+ T cells and NK cells in the TME [84,93,94]. This cascade is expected to convert immunologically cold tumors into hot ones, sensitize tumors to ICB therapies, and induce durable antitumor responses [94]. Indeed, multiple groups including ours have recently shown that acute induction of the cGAS-STING pathway is a promising strategy to enhance ICB efficacy, validated in preclinical models [94,95,96,97]. Additionally, acute STING activation within cancer cells can induce senescence, growth arrest, or apoptosis through canonical interferon-dependent and -independent mechanisms [98,99], further supporting the therapeutic potential of cGAS-STING activation.

However, cGAS-STING signaling may also drive proinflammatory states that paradoxically promote tumor growth and survival, particularly when the pathway is chronically and weakly activated due to chromosomal instability and persistent micronuclei [25,100]. Ahn and colleagues reported that tumor development induced by genotoxic stress with 7,12-dimethylbenz(a)anthracene (DMBA) treatment is efficiently suppressed in STING-/- mice, implicating the direct contribution of STING to tumorigenesis [55]. In addition, Bakhoum et al. reported that cytoplasmic DNA derived from unstable chromatin activates noncanonical NF-kB in a STING-dependent, but TBK-1-independent manner, thereby promoting metastasis of breast cancer cells [62], further indicating that cGAS-STING activation in cancer cells can be sometimes tumor-promoting. Chronic or sustained STING activation leads to persistent type I IFN and inflammatory cytokine release, which fosters an immunosuppressive milieu characterized by regulatory T cell recruitment, myeloid-derived suppressor cell expansion, and upregulation of immune checkpoint molecules such as PD-L1, all of which have potentials to support tumor growth and survival [101,102]. Therefore, the timing and magnitude of STING activation are critical determinants: acute, robust activation in APCs is desirable for antitumor immunity, whereas prolonged, low-level activation may drive immune exhaustion and tumor progression.

## 5. Development and Challenges of STING Agonists for Cancer Therapy

### 5.1. Evolution of STING Agonists: From FAA to Clinical Candidates

Because acute activation of STING in APCs is a promising means to induce antitumor immunity in the TME, direct activation of the pathway by intratumoral injection of STING agonists has been tested and shown impressive potential in preclinical models, indicating a rational for STING agonists in cancer treatment [103,104,105]. Earlier anticancer compounds that induced type I interferon expression and showed antitumor effects included flavone-8-acetic acid (FAA) and its derivative, 5,6-dimethylxanthenone-4 acetic acid (DMXAA) [106]. At the time, STING had not been identified, so researchers observed that FAA and DMXAA disrupted the vasculatures of tumors and reduced tumor burden in mice, without knowing that these chemicals were STING agonists [107]. However, FAA and DMXAA were unimpressive in clinical trials [108]. Later, it was shown that while FAA and DMXAA do bind to mouse STING, these chemicals do not bind to human STING [109]; thus, future STING agonists were designed and developed to target specifically the human protein.

Currently, multiple classes of human STING agonists are in development, categorized according to their molecular structures and mechanisms of action(Table 1): cyclic dinucleotide (CDN) analogs, that mimic natural cyclic GAMP binding to STING; non-CDN STING agonists, that are structurally different from the CDN analogs but also activate STING via a different mechanism; CDN-infused exosomes, which increase the efficiency of CDN getting into cells; bacterial engineered vectors, which utilize modified bacteria that preferentially grows in the tumor environment and release STING agonists to activate immune responses; small molecule–nucleic acid hybrids, that merge the stability of small molecules with the targeting and efficiency of nucleic acids; and undisclosed types of STING agonists, which lack structural information and mechanisms of action but are still studied in clinical trials [110]. Each category has its advantages and limitations. In Phase I clinical trials, the goal is to obtain data for safety, tolerability, pharmacodynamics and pharmacokinetics, and antitumor efficiency; obtaining the correct dosage has been a huge unresolved obstacle as too little STING agonists will have no effect and too much STING agonists can cause extreme toxicities like systemic inflammation, autoimmunity, and cytokine storm, leaving a narrow window for the therapeutic dose [106]. There is also ongoing debate as to which populations of the cells should be induced or stimulated, STING in cancer cells or APCs, immune cells [41,111].

### 5.2. Translating STING Agonists: Clinical Challenges and Solutions

The translation of STING agonists into safe and effective therapies faces several hurdles. First, the instability of agonists such as CDN analogs has limited their utility [129]. Ectonucleotide pyrophosphatases (ENPPs) cleave and inactivate CDNs. Second, systemic administration of potent STING agonists risks excessive inflammation and cytokine-release syndrome [110], so delivery strategies must balance efficacy and toxicity. Intratumoral injection has been employed to limit systemic exposure, but it is impractical for inaccessible or metastatic lesions. Pan and colleagues identified MSA-2, an orally available non-CDN STING agonist that produced tumor regression in mice bearing MC38 tumors with a well-tolerated regimen [130]. Unlike CDNs, non-CDN MSA-2 does not get degraded by ENPPs and better penetrates the cell membrane, and may bind better to some variants of STING alleles; they further showed that the acidified condition of TME contributes to the specificity of MSA-2 activity in tumors, not in normal tissues [130]. Targeted delivery systems, including nanoparticles and antibody conjugates, may also help overcome these two challenges.

Third, STING activation by agonists can provoke protumorigenic reactions in the TME. For example, a recent report from Song et al. showed that STING agonists strongly induce cell-intrinsic expression of PD-L1 in tumor monocytes, which causes resistance to immunotherapy [131]; the authors also found that TLR2 activation can rewire STING signaling to reduce PD-L1 in these monocytes, providing a rational for combination strategies with TLR2 agonists [131]. Thus, understanding the mechanism in which acute STING activation reshapes the TME is essential to mitigate such protumorigenic effects and enable effective immune cell infiltration and function.

Fourth, STING allelic variation complicates clinical translation: single nucleotide polymorphisms generate receptor variants (e.g., R232H) with reduced ligand binding [40,132,133]. Gain-of-function STING alleles also exist and could predispose to autoimmunity [134], so dosing strategies may need to be tailored to STING genotype. Predictive biomarkers for STING agonist efficacy are still limited. STING genotypes may guide agonist selection, but intact downstream of the pathway (e.g., TBK1, IRF3, interferon response genes) is also required. Another necessary component is a competent immune system where APCs and CD8+ T cells are functional. Tumors with low baseline STING activity typically show poor immune infiltration and weak responses to anti-PD-1. By increasing STING activation via delivering STING agonists into the tumor, the release of type I IFNs recruits APCs and T cells and cause inflammation in the tumor as well. The inflammation increases the expression of PD-L1 on tumor cells, which now become more responsive to anti-PD1 treatment [135,136].

Currently, multiple STING agonists are being tested in clinical trials against advanced solid tumors or refractory cancers, with most STING agonists in Phase I trial, a few in Phase II, and none having reached Phase III due to limited durable efficacy [106]. Continued mechanistic and translational studies focused on TME context, temporal dynamics of STING signaling, and targeted delivery will be key to advancing STING-based therapies.

## 6. Discussion

Our review highlights the dualistic nature of cGAS-STING signaling in metastatic progression and dormancy: in general, acute and robust activation in antigen-presenting cells promotes antitumor immunity and can suppress metastatic outgrowth [93,94], whereas chronic and low-level cancer-intrinsic activation often drives noncanonical NF-κB signaling, immunosuppression, and prometastatic phenotypes [25,100]. These context-dependent outcomes underscore the importance of cell type, activation kinetics, and downstream pathway engagement in determining net biological effects. Mechanistically, the balance between type I IFN-driven immune stimulation and NF-κB-mediated inflammatory programs appears to be a critical determinant of whether STING signaling restrains or facilitates metastasis, and emerging evidence implicates intercellular cGAMP transfer, epigenetic regulation of pathway components, and tumor microenvironment composition as key modulators of this balance [92,119,120].

Translating STING biology into effective therapies will require strategies that maximize acute antitumor immunity while minimizing chronic, tumor-promoting inflammation and autoimmunity. Rational approaches include optimized delivery modalities (e.g., targeted or local administration) [41,106,110,111], patient stratification by STING pathway integrity and genotype, and combination regimens that counteract downstream immunosuppressive consequences (e.g., NF-κB modulators, or agents that limit MDSC recruitment). Future work should prioritize spatiotemporal characterization of STING activation in metastatic niches, identify predictive biomarkers of response and toxicity, and employ preclinical models that recapitulate dormancy and metastatic microenvironments to guide safe and effective clinical translation.

## 7. Conclusions

The cGAS-STING pathway plays context-dependent and multifaceted roles in cancer metastasis and dormancy, with its effects determined by the magnitude and duration of activation as well as the cell type in which the pathway is activated. While acute and strong cGAS-STING activation can enhance antitumor immune surveillance and suppress metastatic outgrowth, chronic and weak signaling may promote inflammation, immune suppression, and metastatic progression. Therefore, a better understanding of the spatial and temporal regulation of cGAS-STING signaling will be essential for developing therapeutic strategies that maximize its tumor-suppressive effects while minimizing its tumor-promoting consequences.

## Figures and Tables

**Table 1:** Major classes of STING agonists.

Current Classes of STING Agonists	Mechanisms	STING AGONISTS in Clinical Trials	Ref. No.
Cyclic dinucleotide (CDN) analogs	Mimicking natural cGAMP	MK-1454, ADU-S100(MIW815), BMS-986301, BI-1387446, TAK-676	[112] [113,114] [115] [116] [117]
Non-CDN agonists	Directly binding to STING	SNX281, HG-381, GSK3745417, E-7766	[118] [119] [120] [121]
CDN-infused exosomes	Exosomes delivering CDNs	exoSTING	[122]
Bacterial engineered vectors	Bacterial producing CDNs	SYNB1891	[123]
Small molecule–nucleic acid hybrids	Increasing the molecular stability	SB 11285	[124,125]
Undisclosed type STING agonist	Undisclosed mechanism	MK-2118, XMT-2056	[126,127] [128]

## Data Availability

No new data were created or analyzed in this study.

## Abbreviations

The following abbreviations are used in this manuscript:

APCs	antigen-presenting cells
CAFs	cancer-associated fibroblasts
CDN	cyclic dinucleotide
cGAS	cyclic GMP-AMP synthase
DMBA	7,12-dimethylbenz(a)anthracene
DMXAA	5,6-dimethylxanthenone-4-acetic acid
ECM	extracellular matrix
EMT	Epithelial–Mesenchymal Transition
FAA	flavone-8-acetic acid
ICB	immune checkpoint blockade
MDSC	Myeloid-Derived Suppressor Cell
MET	Mesenchymal–Epithelial Transition
NK	natural killer
STING	stimulator of interferon genes
TME	tumor microenvironment

## References

[1] Hanahan D. (2022). Hallmarks of Cancer: New Dimensions. Cancer Discov..

[2] Hanahan D., Weinberg R.A. (2011). Hallmarks of cancer: The next generation. Cell.

[3] Fidler I.J. (2003). The pathogenesis of cancer metastasis: The ‘seed and soil’ hypothesis revisited. Nat. Rev. Cancer.

[4] Valastyan S., Weinberg R.A. (2011). Tumor Metastasis: Molecular Insights and Evolving Paradigms. Cell.

[5] Nieto M.A., Huang R.Y.-J., Jackson R.A., Thiery J.P. (2016). EMT: 2016. Cell.

[6] Thiery J.P. (2002). Epithelial–mesenchymal transitions in tumour progression. Nat. Rev. Cancer.

[7] Kang Y., Pantel K. (2013). Tumor Cell Dissemination: Emerging Biological Insights from Animal Models and Cancer Patients. Cancer Cell.

[8] Reymond N., D’Agua B.B., Ridley A.J. (2013). Crossing the endothelial barrier during metastasis. Nat. Rev. Cancer.

[9] Headley M.B., Bins A., Nip A., Roberts E.W., Looney M.R., Gerard A., Krummel M.F. (2016). Visualization of immediate immune responses to pioneer metastatic cells in the lung. Nature.

[10] Palumbo J.S., Talmage K.E., Massari J.V., La Jeunesse C.M., Flick M.J., Kombrinck K.W., Jirousková M., Degen J.L. (2005). Platelets and fibrin(ogen) increase metastatic potential by impeding natural killer cell–mediated elimination of tumor cells. Blood.

[11] Chambers A.F., Groom A.C., MacDonald I.C. (2002). Dissemination and growth of cancer cells in metastatic sites. Nat. Rev. Cancer.

[12] Hüsemann Y., Geigl J.B., Schubert F., Musiani P., Meyer M., Burghart E., Forni G., Eils R., Fehm T., Riethmüller G. (2008). Systemic Spread Is an Early Step in Breast Cancer. Cancer Cell.

[13] Harper K.L., Sosa M.S., Entenberg D., Hosseini H., Cheung J.F., Nobre R., Avivar-Valderas A., Nagi C., Girnius N., Davis R.J. (2016). Mechanism of early dissemination and metastasis in Her2^+^ mammary cancer. Nature.

[14] Hosseini H., Obradović M.M.S., Hoffmann M., Harper K.L., Sosa M.S., Werner-Klein M., Nanduri S.L.K., Werno C., Ehrl C., Maneck M. (2016). Early dissemination seeds metastasis in breast cancer. Nature.

[15] Sosa M.S., Bragado P., Aguirre-Ghiso J.A. (2014). Mechanisms of disseminated cancer cell dormancy: An awakening field. Nat. Rev. Cancer.

[16] Aguirre-Ghiso J.A. (2007). Models, mechanisms and clinical evidence for cancer dormancy. Nat. Rev. Cancer.

[17] Ghajar C.M. (2015). Metastasis prevention by targeting the dormant niche. Nat. Rev. Cancer.

[18] Kienast Y., von Baumgarten L., Fuhrmann M., Klinkert W.E.F., Goldbrunner R., Herms J., Winkler F. (2009). Real-time imaging reveals the single steps of brain metastasis formation. Nat. Med..

[19] Piskounova E., Agathocleous M., Murphy M.M., Hu Z., Huddlestun S.E., Zhao Z., Leitch A.M., Johnson T.M., DeBerardinis R.J., Morrison S.J. (2015). Oxidative stress inhibits distant metastasis by human melanoma cells. Nature.

[20] Tubbs A., Nussenzweig A. (2017). Endogenous DNA Damage as a Source of Genomic Instability in Cancer. Cell.

[21] Jackson S.P., Bartek J. (2009). The DNA-damage response in human biology and disease. Nature.

[22] Halazonetis T.D., Gorgoulis V.G., Bartek J. (2008). An Oncogene-Induced DNA Damage Model for Cancer Development. Science.

[23] Bakhoum S.F., Cantley L.C. (2018). The Multifaceted Role of Chromosomal Instability in Cancer and Its Microenvironment. Cell.

[24] Yao Y., Dai W. (2014). Genomic Instability and Cancer. J. Carcinog. Mutagen..

[25] Li T., Chen Z.J. (2018). The cGAS–cGAMP–STING pathway connects DNA damage to inflammation, senescence, and cancer. J. Exp. Med..

[26] Zhang X., Bai X.-C., Chen Z.J. (2020). Structures and Mechanisms in the cGAS-STING Innate Immunity Pathway. Immunity.

[27] Chen Q., Sun L., Chen Z.J. (2016). Regulation and function of the cGAS–STING pathway of cytosolic DNA sensing. Nat. Immunol..

[28] Shang G., Zhang C., Chen Z.J., Bai X.-C., Zhang X. (2019). Cryo-EM structures of STING reveal its mechanism of activation by cyclic GMP–AMP. Nature.

[29] Mukai K., Konno H., Akiba T., Uemura T., Waguri S., Kobayashi T., Barber G.N., Arai H., Taguchi T. (2016). Activation of STING requires palmitoylation at the Golgi. Nat. Commun..

[30] Hansen A.L., Buchan G.J., Rühl M., Mukai K., Salvatore S.R., Ogawa E., Andersen S.D., Iversen M.B., Thielke A.L., Gunderstofte C. (2018). Nitro-fatty acids are formed in response to virus infection and are potent inhibitors of STING palmitoylation and signaling. Proc. Natl. Acad. Sci. USA.

[31] Matsumoto K., Ni S., Arai H., Toyama T., Saito Y., Suzuki T., Dohmae N., Mukai K., Taguchi T. (2023). A non-nucleotide agonist that binds covalently to cysteine residues of STING. Cell Struct. Funct..

[32] Takahashi K., Niki T., Ogawa E., Fumika K., Nishioka Y., Sawa M., Arai H., Mukai K., Taguchi T. (2021). A cell-free assay implicates a role of sphingomyelin and cholesterol in STING phosphorylation. Sci. Rep..

[33] Kemmoku H., Kuchitsu Y., Mukai K., Taguchi T. (2022). Specific association of TBK1 with the trans-Golgi network following STING stimulation. Cell Struct. Funct..

[34] Kemmoku H., Takahashi K., Mukai K., Mori T., Hirosawa K.M., Kiku F., Uchida Y., Kuchitsu Y., Nishioka Y., Sawa M. (2024). Single-molecule localization microscopy reveals STING clustering at the trans-Golgi network through palmitoylation-dependent accumulation of cholesterol. Nat. Commun..

[35] Zhang B., Xu P., Ablasser A. (2026). Regulation of the CGAS-STING Pathway. Annu. Rev. Immunol..

[36] Shen M., Jiang X., Peng Q., Oyang L., Ren Z., Wang J., Peng M., Zhou Y., Deng X., Liao Q. (2025). The cGAS–STING pathway in cancer immunity: Mechanisms, challenges, and therapeutic implications. J. Hematol. Oncol..

[37] Shoji T., Shinojima A., Kishimoto T., Sato K., Ikegami N., Yumoto E., Shindo R., Uchida Y., Kusumi S., Koga D. (2026). A PI(3,5)P2/CHMP4B axis on lysosomes is essential for microautophagic degradation of STING. Nat. Commun..

[38] Kuchitsu Y., Mukai K., Uematsu R., Takaada Y., Shinojima A., Shindo R., Shoji T., Hamano S., Ogawa E., Sato R. (2023). STING signalling is terminated through ESCRT-dependent microautophagy of vesicles originating from recycling endosomes. Nat. Cell Biol..

[39] Tan X., Sun L., Chen J., Chen Z.J. (2018). Detection of Microbial Infections Through Innate Immune Sensing of Nucleic Acids. Annu. Rev. Microbiol..

[40] Koide S., Yumoto E., Nakayama J., Higashiyama S., Kuchitsu Y., Taguchi T. (2025). Cell biological insights into human STING variants. Cell Struct. Funct..

[41] Jiang M., Chen P., Wang L., Li W., Chen B., Liu Y., Wang H., Zhao S., Ye L., He Y. (2020). cGAS-STING, an important pathway in cancer immunotherapy. J. Hematol. Oncol..

[42] Xia T., Konno H., Barber G.N. (2016). Recurrent Loss of STING Signaling in Melanoma Correlates with Susceptibility to Viral Oncolysis. Cancer Res..

[43] Xia T., Konno H., Ahn J., Barber G.N. (2016). Deregulation of STING Signaling in Colorectal Carcinoma Constrains DNA Damage Responses and Correlates With Tumorigenesis. Cell Rep..

[44] Dou Z., Ghosh K., Vizioli M.G., Zhu J., Sen P., Wangensteen K.J., Simithy J., Lan Y., Lin Y., Zhou Z. (2017). Cytoplasmic chromatin triggers inflammation in senescence and cancer. Nature.

[45] Yang H., Wang H., Ren J., Chen Q., Chen Z.J. (2017). cGAS is essential for cellular senescence. Proc. Natl. Acad. Sci. USA.

[46] Ranoa D.R.E., Widau R.C., Mallon S., Parekh A.D., Nicolae C.M., Huang X., Bolt M.J., Arina A., Parry R., Kron S.J. (2019). STING Promotes Homeostasis via Regulation of Cell Proliferation and Chromosomal Stability. Cancer Res..

[47] Falahat R., Berglund A., Putney R.M., Perez-Villarroel P., Aoyama S., Pilon-Thomas S., Barber G.N., Mulé J.J. (2021). Epigenetic reprogramming of tumor cell–intrinsic STING function sculpts antigenicity and T cell recognition of melanoma. Proc. Natl. Acad. Sci. USA.

[48] DeMaria O., Cornen S., Daëron M., Morel Y., Medzhitov R., Vivier E. (2019). Harnessing innate immunity in cancer therapy. Nature.

[49] Takashima K., Takeda Y., Oshiumi H., Shime H., Okabe M., Ikawa M., Matsumoto M., Seya T. (2016). STING in tumor and host cells cooperatively work for NK cell-mediated tumor growth retardation. Biochem. Biophys. Res. Commun..

[50] Yan X., Yao C., Fang C., Han M., Gong C., Hu D., Shen W., Wang L., Li S., Zhu S. (2022). Rocaglamide promotes the infiltration and antitumor immunity of NK cells by activating cGAS-STING signaling in non-small cell lung cancer. Int. J. Biol. Sci..

[51] Mackenzie K.J., Carroll P., Martin C.-A., Murina O., Fluteau A., Simpson D.J., Olova N., Sutcliffe H., Rainger J.K., Leitch A. (2017). cGAS surveillance of micronuclei links genome instability to innate immunity. Nature.

[52] Al-Adayleh T.Y.M., Ahmad W., Rahiman S.S.F., Murugaiyah V., Mohamed R., Afzal S., Al-Ramamneh R.S., Attiq A. (2026). cGAS-STING Signalling in the tumour microenvironment: Implications for immune surveillance and therapeutic strategies. Int. Immunopharmacol..

[53] Marinello J., Arleo A., Russo M., Delcuratolo M., Ciccarelli F., Pommier Y., Capranico G. (2022). Topoisomerase I poison-triggered immune gene activation is markedly reduced in human small-cell lung cancers by impairment of the cGAS/STING pathway. Br. J. Cancer.

[54] Huang M., Cha Z., Liu R., Lin M., Gafoor N.A., Kong T., Ge F., Chen W. (2024). Enhancing immunotherapy outcomes by targeted remodeling of the tumor microenvironment via combined cGAS-STING pathway strategies. Front. Immunol..

[55] Ahn J., Xia T., Konno H., Konno K., Ruiz P., Barber G.N. (2014). Inflammation-driven carcinogenesis is mediated through STING. Nat. Commun..

[56] Du J.-M., Qian M.-J., Yuan T., Chen R.-H., He Q.-J., Yang B., Ling Q., Zhu H. (2022). cGAS and cancer therapy: A double-edged sword. Acta Pharmacol. Sin..

[57] Huang D., Rezaeian A.-H., Wang J., Qi Y., Chen H., Inuzuka H., Wei W. (2024). Targeting the PRMT1-cGAS-STING signaling pathway to enhance the anti-tumor therapeutic efficacy. J. Cancer Biol..

[58] Yue B., Gao W., Lovell J.F., Jin H., Huang J. (2025). The cGAS-STING pathway in cancer immunity: Dual roles, therapeutic strategies, and clinical challenges. Essays Biochem..

[59] Shen R., Liu D., Wang X., Guo Z., Sun H., Song Y., Wang D. (2022). DNA Damage and Activation of cGAS/STING Pathway Induce Tumor Microenvironment Remodeling. Front. Cell Dev. Biol..

[60] Khan S.U., Halawi M.H., Almehmadi M., Taha R., Ahmed A.E., Alfaifi M.Y., Shati A.A., Elbehairi S.E.I., Ahmad S., Alanazi Y.F. (2026). Innate immune recognition and microenvironmental reprogramming in HPV-induced cervical cancer: From pattern recognition receptor activation to immune tolerance disruption. Front. Cell. Infect. Microbiol..

[61] Samson N., Ablasser A. (2022). The cGAS–STING pathway and cancer. Nat. Cancer.

[62] Bakhoum S.F., Ngo B., Laughney A.M., Cavallo J.-A., Murphy C.J., Ly P., Shah P., Sriram R.K., Watkins T.B.K., Taunk N.K. (2018). Chromosomal instability drives metastasis through a cytosolic DNA response. Nature.

[63] Beernaert B., Jady-Clark R.L., Shah P., Ramon-Gil E., Lawson N.M., Brodtman Z.D., Tagore S., Stihler F., Carter A.S., Clarke S. (2026). Chromosomal instability shapes the tumor microenvironment of esophageal adenocarcinoma via a cGAS–chemokine–myeloid axis. Sci. Adv..

[64] Chen Q., Boire A., Jin X., Valiente M., Er E.E., Lopez-Soto A., Jacob L.S., Patwa R., Shah H., Xu K. (2016). Carcinoma–astrocyte gap junctions promote brain metastasis by cGAMP transfer. Nature.

[65] Zhang H., Qian W., Li M., Zou X., Chen X., Miao K., Zhao M., Xu M., Dong J., Wang J. (2026). SEL1L3 suppresses colorectal cancer cell growth and metastasis by preventing endoplasmic reticulum-associated degradation of STING. Cell Death Dis..

[66] Massagué J., Obenauf A.C. (2016). Metastatic colonization by circulating tumour cells. Nature.

[67] Risson E., Nobre A.R., Maguer-Satta V., Aguirre-Ghiso J.A. (2020). The current paradigm and challenges ahead for the dormancy of disseminated tumor cells. Nat. Cancer.

[68] Massagué J., Ganesh K. (2021). Metastasis-Initiating Cells and Ecosystems. Cancer Discov..

[69] Wang Z., Elbanna Y., Godet I., Gan S., Peters L., Lampe G., Chen Y., Xavier J., Huse M., Massagué J. (2026). TGFβ induces an atypical EMT to evade immune mechanosurveillance in lung adenocarcinoma dormant metastasis. Nat. Cancer.

[70] Goddard E.T., Linde M.H., Srivastava S., Klug G., Shabaneh T.B., Iannone S., Grzelak C.A., Marsh S., Riggio A.I., Shor R.E. (2024). Immune evasion of dormant disseminated tumor cells is due to their scarcity and can be overcome by T cell immunotherapies. Cancer Cell.

[71] Pommier A., Anaparthy N., Memos N., Kelley Z.L., Gouronnec A., Yan R., Auffray C., Albrengues J., Egeblad M., Iacobuzio-Donahue C.A. (2018). Unresolved endoplasmic reticulum stress engenders immune-resistant, latent pancreatic cancer metastases. Science.

[72] Malladi S., Macalinao D.G., Jin X., He L., Basnet H., Zou Y., De Stanchina E., Massagué J. (2016). Metastatic Latency and Immune Evasion through Autocrine Inhibition of WNT. Cell.

[73] Hu J., Sánchez-Rivera F.J., Wang Z., Johnson G.N., Ho Y.-J., Ganesh K., Umeda S., Gan S., Mujal A.M., Delconte R.B. (2023). STING inhibits the reactivation of dormant metastasis in lung adenocarcinoma. Nature.

[74] Bushnell G.G., Sharma D., Wilmot H.C., Zheng M., Fashina T.D., Hutchens C.M., Osipov S., Burness M., Wicha M.S. (2024). Natural Killer Cell Regulation of Breast Cancer Stem Cells Mediates Metastatic Dormancy. Cancer Res..

[75] Jakab M., Lee K.H., Uvarovskii A., Ovchinnikova S., Kulkarni S.R., Jakab S., Rostalski T., Spegg C., Anders S., Augustin H.G. (2024). Lung endothelium exploits susceptible tumor cell states to instruct metastatic latency. Nat. Cancer.

[76] Aliazis K., Christofides A., Shah R., Yeo Y.Y., Jiang S., Charest A., Boussiotis V.A. (2025). The tumor microenvironment’s role in the response to immune checkpoint blockade. Nat. Cancer.

[77] Pitt J.M., Marabelle A., Eggermont A., Soria J.-C., Kroemer G., Zitvogel L. (2016). Targeting the tumor microenvironment: Removing obstruction to anticancer immune responses and immunotherapy. Ann. Oncol..

[78] de Visser K.E., Joyce J.A. (2023). The evolving tumor microenvironment: From cancer initiation to metastatic outgrowth. Cancer Cell.

[79] Gajewski T.F., Schreiber H., Fu Y.-X. (2013). Innate and adaptive immune cells in the tumor microenvironment. Nat. Immunol..

[80] Lamplugh Z.L., Wellhausen N., June C.H., Fan Y. (2025). Microenvironmental regulation of solid tumour resistance to CAR T cell therapy. Nat. Rev. Immunol..

[81] Galassi C., Chan T.A., Vitale I., Galluzzi L. (2024). The hallmarks of cancer immune evasion. Cancer Cell.

[82] Jiang X., Wang J., Deng X., Xiong F., Zhang S., Gong Z., Li X., Cao K., Deng H., He Y. (2020). The role of microenvironment in tumor angiogenesis. J. Exp. Clin. Cancer Res..

[83] Schoggins J.W., MacDuff D.A., Imanaka N., Gainey M.D., Shrestha B., Eitson J.L., Mar K.B., Richardson R.B., Ratushny A.V., Litvak V. (2014). Pan-viral specificity of IFN-induced genes reveals new roles for cGAS in innate immunity. Nature.

[84] Dvorkin S., Cambier S., Volkman H.E., Stetson D.B. (2024). New frontiers in the cGAS-STING intracellular DNA-sensing pathway. Immunity.

[85] Crossley M.P., Song C., Bocek M.J., Choi J.-H., Kousouros J.N., Sathirachinda A., Lin C., Brickner J.R., Bai G., Lans H. (2022). R-loop-derived cytoplasmic RNA–DNA hybrids activate an immune response. Nature.

[86] Ou L., Zhang A., Cheng Y., Chen Y. (2021). The cGAS-STING Pathway: A Promising Immunotherapy Target. Front. Immunol..

[87] Woo S.-R., Fuertes M.B., Corrales L., Spranger S., Furdyna M.J., Leung M.Y.K., Duggan R., Wang Y., Barber G.N., Fitzgerald K.A. (2014). STING-Dependent Cytosolic DNA Sensing Mediates Innate Immune Recognition of Immunogenic Tumors. Immunity.

[88] Dai E., Zhu Z., Wahed S., Qu Z., Storkus W.J., Guo Z.S. (2021). Epigenetic modulation of antitumor immunity for improved cancer immunotherapy. Mol. Cancer.

[89] Kwon J., Bakhoum S.F. (2020). The Cytosolic DNA-Sensing cGAS–STING Pathway in Cancer. Cancer Discov..

[90] Kanoda R., Nakajima S., Okayama H., Kaneta A., Chida S., Matsumoto T., Saito K., Kikuchi T., Maruyama Y., Suzuki H. (2025). Downregulation of cGAS/STING expression in tumor cells by cancer-associated fibroblasts in colorectal cancer. Sci. Rep..

[91] Ringham O.R., Rivera M., Loffredo L.F., Ozsoy M.A., Healy C.M., Cheng M.F., Jin Y., Chen N., Santos-Alexis K.d.L., Azizi E. (2026). A novel CAF population coordinates hyper-suppressive regulatory T cell recruitment and localization in lung cancer. Nat. Immunol..

[92] Simon T., Salhia B. (2022). Cancer-Associated Fibroblast Subpopulations With Diverse and Dynamic Roles in the Tumor Microenvironment. Mol. Cancer Res..

[93] Fahey C.G., Cordova A.F., Gedeon P.C., Barbie D.A. (2025). Targeting STING to generate therapeutic anti-tumor immunity. Cancer Cell.

[94] Vanpouille-Box C., Alard A., Aryankalayil M.J., Sarfraz Y., Diamond J.M., Schneider R.J., Inghirami G., Coleman C.N., Formenti S.C., Demaria S. (2017). DNA exonuclease Trex1 regulates radiotherapy-induced tumour immunogenicity. Nat. Commun..

[95] Shen J.Z., Qiu Z., Wu Q., Finlay D., Garcia G., Sun D., Rantala J., Barshop W., Hope J.L., Gimple R.C. (2021). FBXO44 promotes DNA replication-coupled repetitive element silencing in cancer cells. Cell.

[96] Tani T., Mathsyaraja H., Campisi M., Li Z.-H., Haratani K., Fahey C.G., Ota K., Mahadevan N.R., Shi Y., Saito S. (2024). *TREX1* Inactivation Unleashes Cancer Cell STING–Interferon Signaling and Promotes Antitumor Immunity. Cancer Discov..

[97] Murayama T., Nakayama J., Jiang X., Miyata K., Morris A.D., Cai K.Q., Prasad R.M., Ma X., Efimov A., Belani N. (2024). Targeting DHX9 Triggers Tumor-Intrinsic Interferon Response and Replication Stress in Small Cell Lung Cancer. Cancer Discov..

[98] Hopfner K.-P., Hornung V. (2020). Molecular mechanisms and cellular functions of cGAS–STING signalling. Nat. Rev. Mol. Cell Biol..

[99] Liu S., Guan W. (2018). STING Signaling Promotes Apoptosis, Necrosis, and Cell Death: An Overview and Update. Mediat. Inflamm..

[100] Decout A., Katz J.D., Venkatraman S., Ablasser A. (2021). The cGAS–STING pathway as a therapeutic target in inflammatory diseases. Nat. Rev. Immunol..

[101] Boukhaled G.M., Harding S., Brooks D.G. (2020). Opposing Roles of Type I Interferons in Cancer Immunity. Annu. Rev. Pathol. Mech. Dis..

[102] Benci J.L., Johnson L.R., Choa R., Xu Y., Qiu J., Zhou Z., Xu B., Ye D., Nathanson K.L., June C.H. (2019). Opposing Functions of Interferon Coordinate Adaptive and Innate Immune Responses to Cancer Immune Checkpoint Blockade. Cell.

[103] Najem H., Lea S.T., Tripathi S., Hurley L., Chen C.-H., William I., Sooreshjani M., Bowie M., Hartley G., Dussold C. (2024). STING agonist 8803 reprograms the immune microenvironment and increases survival in preclinical models of glioblastoma. J. Clin. Investig..

[104] Ohkuri T., Ghosh A., Kosaka A., Zhu J., Ikeura M., David M., Watkins S.C., Sarkar S.N., Okada H. (2014). STING Contributes to Antiglioma Immunity via Triggering Type I IFN Signals in the Tumor Microenvironment. Cancer Immunol. Res..

[105] Corrales L., Glickman L.H., McWhirter S.M., Kanne D.B., Sivick K.E., Katibah G.E., Woo S.-R., Lemmens E., Banda T., Leong J.J. (2015). Direct Activation of STING in the Tumor Microenvironment Leads to Potent and Systemic Tumor Regression and Immunity. Cell Rep..

[106] Le Naour J., Zitvogel L., Galluzzi L., Vacchelli E., Kroemer G. (2020). Trial watch: STING agonists in cancer therapy. OncoImmunology.

[107] Jameson M.B., Sharp D.M., Sissingh J.I., Hogg C.R., Thompson P.I., McKeage M.J., Jeffery M., Waller S., Acton G., Green C. (2009). Transient Retinal Effects of 5,6-Dimethylxanthenone-4-acetic Acid (DMXAA, ASA404), an Antitumor Vascular-Disrupting Agent in Phase I Clinical Trials. Investig. Opthalmology Vis. Sci..

[108] Kim S., Li L., Maliga Z., Yin Q., Wu H., Mitchison T.J. (2013). Anticancer Flavonoids Are Mouse-Selective STING Agonists. ACS Chem. Biol..

[109] Conlon J., Burdette D.L., Sharma S., Bhat N., Thompson M., Jiang Z., Rathinam V.A.K., Monks B., Jin T., Xiao T.S. (2013). Mouse, but not Human STING, Binds and Signals in Response to the Vascular Disrupting Agent 5,6-Dimethylxanthenone-4-Acetic Acid. J. Immunol..

[110] Wang B., Yu W., Jiang H., Meng X., Tang D., Liu D. (2024). Clinical applications of STING agonists in cancer immunotherapy: Current progress and future prospects. Front. Immunol..

[111] Wang Y., Luo J., Alu A., Han X., Wei Y., Wei X. (2020). cGAS-STING pathway in cancer biotherapy. Mol. Cancer.

[112] Harrington K., Brody J., Ingham M., Strauss J., Cemerski S., Wang M., Tse A., Khilnani A., Marabelle A., Golan T. (2018). Preliminary results of the first-in-human (FIH) study of MK-1454, an agonist of stimulator of interferon genes (STING), as monotherapy or in combination with pembrolizumab (pembro) in patients with advanced solid tumors or lymphomas. Ann. Oncol..

[113] Meric-Bernstam F., Sandhu S.K., Hamid O., Spreafico A., Kasper S., Dummer R., Shimizu T., Steeghs N., Lewis N., Talluto C.C. (2019). Phase Ib study of MIW815 (ADU-S100) in combination with spartalizumab (PDR001) in patients (pts) with advanced/metastatic solid tumors or lymphomas. J. Clin. Oncol..

[114] Meric-Bernstam F., Sweis R.F., Hodi F.S., Messersmith W.A., Andtbacka R.H.I., Ingham M., Lewis N., Chen X., Pelletier M., Chen X. (2022). Phase I Dose-Escalation Trial of MIW815 (ADU-S100), an Intratumoral STING Agonist, in Patients with Advanced/Metastatic Solid Tumors or Lymphomas. Clin. Cancer Res..

[115] Li K., Wang J., Zhang R., Zhou J., Espinoza B., Niu N., Wang J., Jurcak N., Rozich N., Osipov A. (2024). Overcome the challenge for intratumoral injection of STING agonist for pancreatic cancer by systemic administration. J. Hematol. Oncol..

[116] Harrington K., Parkes E., Weiss J., Ingham M., Cervantes A., Calvo E., Klinkhardt U., Sikken P., Schmohl M., Garralda E. (2021). Abstract CT217: Phase I, first-in-human trial evaluating BI 1387446 (STING agonist) alone and in combination with ezabenlimab (BI 754091; anti-PD-1) in solid tumors. Cancer Res..

[117] Carideo Cunniff E., Sato Y., Mai D., Appleman V.A., Iwasaki S., Kolev V., Matsuda A., Shi J., Mochizuki M., Yoshikawa M. (2022). TAK-676: A Novel Stimulator of Interferon Genes (STING) Agonist Promoting Durable IFN-Dependent Antitumor Immunity in Preclinical Studies. Cancer Res. Commun..

[118] Wang J., Falchook G., Nabhan S., Kulkarni M., Sandy P., Dosunmu O., Gardner H., Bendell J., Johnson M. 495 Trial of SNX281, a systemically delivered small molecule STING agonist, in solid tumors and lymphomas. Proceedings of the SITC 36th Anniversary Annual Meeting (SITC 2021) Abstracts.

[119] Fan Y., Feng R., Zhang X., Wang Z.-L., Xiong F., Zhang S., Zhong Z.-F., Yu H., Zhang Q.-W., Zhang Z. (2024). Encoding and display technologies for combinatorial libraries in drug discovery: The coming of age from biology to therapy. Acta Pharm. Sin. B.

[120] Montesinos P., Al-Ali H., Alonso-Dominguez J.M., Jentzsch M., Jongen-Lavrencic M., Martelli M.P., Röllig C., Sica S., Iadevaia R., Yablonski K. (2023). Abstract CT124: A first-in-clinic phase 1 study of GSK3745417 STING agonist in relapsed/refractory acute myeloid leukemia and high-risk myelodysplastic syndrome. Cancer Res..

[121] Endo A., Kim D.-S., Huang K.-C., Hao M.-H., Mathieu S., Choi H.-W., Majumder U., Zhu X., Shen Y., Sanders K. (2019). Abstract 4456: Discovery of E7766: A representative of a novel class of macrocycle-bridged STING agonists (MBSAs) with superior potency and pan-genotypic activity. Cancer Res..

[122] Jang S.C., Economides K.D., Moniz R.J., Sia C.L., Lewis N., McCoy C., Zi T., Zhang K., Harrison R.A., Lim J. (2021). ExoSTING, an extracellular vesicle loaded with STING agonists, promotes tumor immune surveillance. Commun. Biol..

[123] Leventhal D.S., Sokolovska A., Li N., Plescia C., Kolodziej S.A., Gallant C.W., Christmas R., Gao J.-R., James M.J., Abin-Fuentes A. (2020). Immunotherapy with engineered bacteria by targeting the STING pathway for anti-tumor immunity. Nat. Commun..

[124] Challa S.V., Zhou S., Sheri A., Padmanabhan S., Meher G., Gimi R., Schmidt D., Cleary D., Afdhal N., Iyer R. (2017). Preclinical studies of SB 11285, a novel STING agonist for immuno-oncology. J. Clin. Oncol..

[125] Challa S., Ramachandran B., Vijayakrishnan L., Weitzel D., Zhou S., Iyer K. (2020). Abstract B96: Pharmacodynamic studies of SB 11285, a systemically bioavailable STING agonist in orthotopic tumor models. Cancer Immunol. Res..

[126] Lyons T.W., Thaisrivongs D.A., Kuhl N., Chung C.K., Angeles A., Steinhuebel D., Guetschow E., Brunskill A.P.J., Henderson T.J., Cash B.D. (2024). The First GMP Synthesis of MK-2118, a Small Molecule Agonist for Stimulator of Interferon Genes. Org. Process Res. Dev..

[127] Duvall J.R., Bukhalid R.A., Cetinbas N.M., Catcott K.C., Lancaster K., Bentley K.W., Clark S., Clardy S., Collins S.D., Dirksen A. (2022). Abstract 3503: XMT-2056, a HER2-targeted Immunosynthen STING-agonist antibody-drug conjugate, binds a novel epitope of HER2 and shows increased anti-tumor activity in combination with trastuzumab and pertuzumab. Cancer Res..

[128] Soomer-James J., Damelin M., Malli N. (2023). Abstract 4423: XMT-2056, a HER2-targeted STING agonist antibody-drug conjugate, exhibits ADCC function that synergizes with STING pathway activation and contributes to anti-tumor responses. Cancer Res..

[129] Amouzegar A., Chelvanambi M., Filderman J.N., Storkus W.J., Luke J.J. (2021). STING Agonists as Cancer Therapeutics. Cancers.

[130] Pan B.-S., Perera S.A., Piesvaux J.A., Presland J.P., Schroeder G.K., Cumming J.N., Trotter B.W., Altman M.D., Buevich A.V., Cash B. (2020). An orally available non-nucleotide STING agonist with antitumor activity. Science.

[131] Song H., Chen L., Pan X., Shen Y., Ye M., Wang G., Cui C., Zhou Q., Tseng Y., Gong Z. (2025). Targeting tumor monocyte-intrinsic PD-L1 by rewiring STING signaling and enhancing STING agonist therapy. Cancer Cell.

[132] Froechlich G., Finizio A., Napolano A., Amiranda S., De Chiara A., Pagano P., Mallardo M., Leoni G., Zambrano N., Sasso E. (2023). The common H232 STING allele shows impaired activities in DNA sensing, susceptibility to viral infection, and in monocyte cell function, while the HAQ variant possesses wild-type properties. Sci. Rep..

[133] Shoji T., Sato K., Shinojima A., Koide S., Shindo R., Hongo K., Mukai K., Kuchitsu Y., Taguchi T. (2025). A quantitative method to monitor STING degradation with dual-luciferase reporters. Cell Struct. Funct..

[134] Bennion B.G., Croft C.A., Ai T.L., Qian W., Menos A.M., Miner C.A., Frémond M.-L., Doisne J.-M., Andhey P.S., Platt D.J. (2020). STING Gain-of-Function Disrupts Lymph Node Organogenesis and Innate Lymphoid Cell Development in Mice. Cell Rep..

[135] Nakamura T., Sato T., Endo R., Sasaki S., Takahashi N., Sato Y., Hyodo M., Hayakawa Y., Harashima H. (2021). STING agonist loaded lipid nanoparticles overcome anti-PD-1 resistance in melanoma lung metastasis via NK cell activation. J. Immunother. Cancer.

[136] Li T., Zhang W., Niu M., Wu Y., Deng X., Zhou J. (2024). STING agonist inflames the cervical cancer immune microenvironment and overcomes anti-PD-1 therapy resistance. Front. Immunol..

